# Two domain-disrupted hda6 alleles have opposite epigenetic effects on transgenes and some endogenous targets

**DOI:** 10.1038/srep17832

**Published:** 2015-12-15

**Authors:** Shoudong Zhang, Xiangqiang Zhan, Xiaoming Xu, Peng Cui, Jian-Kang Zhu, Yiji Xia, Liming Xiong

**Affiliations:** 1 Department of Biology, Hong Kong Baptist University, Hong Kong, Hong Kong SAR, China; 2 Shanghai Center for Plant Stress Biology, Chinese Academy of Sciences, Shanghai, China; 3 College of life sciences, Nanjing Agricultural University, Nanjing, China; 4 Horticulture and Landscape Architecture, Purdue University, West Lafayette, IN 47907, USA; 5 Division of Biological and Environmental Sciences & Engineering, King Abdullah University of Science and Technology (KAUST), Thuwal, Saudi Arabia

## Abstract

HDA6 is a RPD3-like histone deacetylase. In Arabidopsis, it mediates transgene and some endogenous target transcriptional gene silencing (TGS) via histone deacetylation and DNA methylation. Here, we characterized two *hda6* mutant alleles that were recovered as second-site suppressors of the DNA demethylation mutant *ros1–1*. Although both alleles derepressed *35S::NPTII* and *RD29A::LUC* in the *ros1–1* background, they had distinct effects on the expression of these two transgenes. In accordance to expression profiles of two transgenes, the alleles have distinct opposite methylation profiles on two reporter gene promoters. Furthermore, both alleles could interact *in vitro* and *in vivo* with the DNA methyltransferase1 with differential interactive strength and patterns. Although these alleles accumulated different levels of repressive/active histone marks, DNA methylation but not histone modifications in the two transgene promoters was found to correlate with the level of derepression of the reporter genes between the two *had6* alleles. Our study reveals that mutations in different domains of HDA6 convey different epigenetic status that in turn controls the expression of the transgenes as well as some endogenous loci.

DNA methylation and histone modifications dictate the chromatin state and greatly affect gene expression in animals as well as in plants. Unlike in mammals whose DNA methylation is largely limited to symmetrical CG dinucleotides mediated by the methyltransferases DNMT1 and DNMT3^1^, methylation in Arabidopsis can occur on cytosine in any nucleotide context including symmetrical cytosine (CG and CHG), as well as asymmetrical cytosine (CHH) (H = A, T, and C) sites^2^. Symmetrical cytosine methylation in Arabidopsis is maintained by two DNA methylatransferases, MET1 (a DNMT1 homolog for CG methylation) and CMT3 (for CHG methylation)^3^. These methyltransferases can restore the methylation status to a newly synthesized DNA strand based on the parental strand methylation status with the help of VIM proteins^4^ and the KYP/SUVH4 protein^5^, respectively. On the other hand, asymmetrical cytosine methylation, which is mainly catalyzed by DRM2, would need *de novo* methylation guided by Pol IV and Pol V via the RNA-directed DNA methylation (RdDM) pathway and/or the Pol II-mediated pathway^14^. The plant-specific RdDM pathway uses small interfering RNA (siRNA) to guide the methylation and silencing of transposons, repetitive sequences and certain gene promoters^2^. RdDM involves siRNA biogenesis mediated by DCL3, RDR2, Pol IV, and/or Pol V^7^,^8^,^9^, scaffold RNA production via Pol II, Pol V, DRD1, DMS3 and RDM1^10^,^12^,^13^, and formation of the guiding complex as well as DRM2 recruitment^14^. Besides these components that are directly involved in the RdDM pathway, forward genetic screening also identified other components with profound effects on RdDM, such as RDM4, RDM12, KTF1, and HDA6^15^,^16^,^17^,^18^.

DNA methylation and histone modifications are closely linked processes^19^. The cross-talk between DNA methylation and histone modification was initially observed in suvh39 mutated mice in which the histone methyltransferase mutation caused failure of localization of DNMT3B to the pericentromeric heterochromatin region. As a result, DNA methylation in that region was decreased^20^. In Arabidopsis, mutations in the histone H3K9 methyltransferase KYP decrease not only the accumulation of histone H3 lysine 9 dimethylation (H3K9me2) but also the levels of CHG DNA methylation^6^,^21^. Recently, the structural basis for CMT3-mediated CHG methylation at the H3K9me2-containing nucleosome has been described^5^. Both the BAH domain and the chromodomain of CMT3 were found to bind to H3K9me2-containing nucleosomes where the unmethylated cytosine was converted to methylated cytosine in the CHG context^5^. More recently, the histone acetylase IDM1/ROS4 was found to play a role in active DNA demethylation, a process that eukaryotic cells employ to fine-tune the methylation status to limit the silencing of certain genes^22^,^23^.

The Arabidopsis Repressor of Silence 1 (ROS1) DNA glycosylase/lysase can remove the 5-methyl group from methylcytosine at target sites using a base excision repair mechanism^24^. In the *ros1–1* mutant, the two-linked reporter genes, *35S* promoter driven *NPTII* (*35S::NPTII*) and *RD29A* promoter driven firefly luciferase gene (*LUC*) (*RD29A::LUC*), were both silenced as a result of reduced demethylation in their promoters^25^. Nonetheless, the silencing mechanisms for these two promoters are different. The silencing of the *RD29A::LUC* is entirely dependent on the RdDM pathway, whereas the silencing of *35S::NPTII* is not^26^.

To better understand the mechanisms underlying the silencing of these two reporter genes and the endogenous loci that they represent, we conducted genetic screens for *ros1–1* suppressors^16^. Here we identified two different *hda6* alleles that can release the two reporter genes albeit to different extents. One allele (*hda6–9*) has a stronger effect on the release of the *35S*::*NPTII* transgene while the other allele (*hda6–10*) has a stronger release of the *RD29A::LUC* transgene. We explored the mechanisms that underlie the differential derepression on the two reporter genes between the two *hda6* alleles. Our results showed that the distinct DNA methylation patterns of the reporter gene promoters between the two *hda6* alleles are crucial in determining the reporter gene expression, although the elevated acetylation level caused by loss of deacetylation on the reporter gene promoters also contributes to the expression patterns of the reporter genes.

## Results

### Two *hda6* mutations suppress *ros1*-mediated transcriptional gene silencing and differentially regulate reporter gene expression

**Figure 1:**
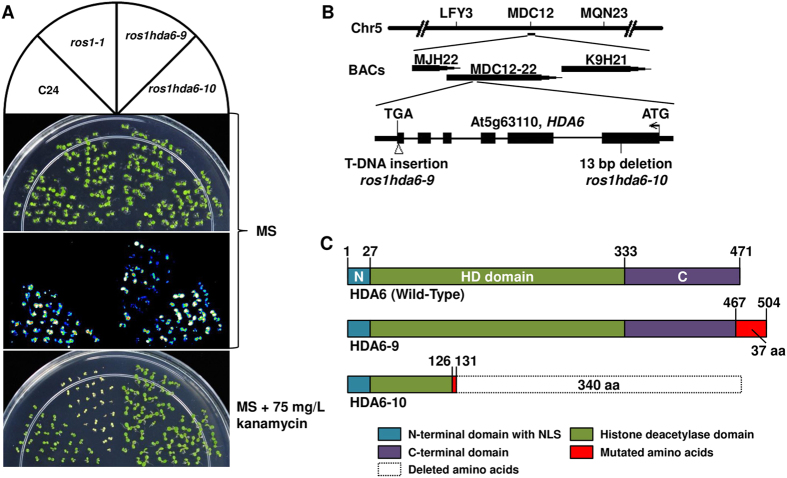
Luminescence and kanamycin resistance phenotypes of *ros1* suppressors. (**A**) WT (*C24-LUC*, harboring the *RD29A::LUC* transgene), *ros1–1*, and the two suppressor mutants, *hda6–9* and *hda6–10,* were either grown on MS agar plates for 7 d and their luminescence was imaged after cold treatment (1 d, 4 °C); or they were grown on MS agar plates supplemented with 75 μg/ml kanamycin and their pictures were taken 10 days after germination. (**B**) Molecular mapping delimited the mutation of *hda6–9* to the interval between the molecular markers MDC12-10 and MDC12-50. (**C**) Schematic structure of the wild type HDA6, hda6–10 and hda6–9 proteins.

The Arabidopsis *repressor of silencing* (*ros1–1*) mutation led to transcriptional silencing of two expressed reporter genes (*35S::NPTII* and *RD29A::LUC*) that were introduced into its wild-type background as a single transfer-DNA (T-DNA)^25^,^27^. In a forward genetic screen for *ros1–1* suppressors, we identified two suppressors of *ros1–1* that releases both the *35S::NPTII* and the *RD29A::LUC* reporter genes (Fig. 1A). Although the mutants were generated by T-DNA mutagenesis, PCR-based methods were unsuccessful in identifying the flanking sequence of the insertion in the first mutant. We thus conducted genetic mapping and the mutation was eventually narrowed down to an interval delimited by two molecular markers MDC12–10 and MDC12–50 (Fig. 1B). By sequencing the genes within the interval, we found that there was a T-DNA inserted at 12 bp before the stop codon of the *HDA6* gene, which encodes the histone deacetylase 6 (HDA6) protein. This insertion would replace the last 4 amino acids of the predicated protein with 37 new amino acids before encountering a new stop codon. Since *hda6* mutants in different background have been numbered up to *hda6–6* (*axe1–5*), we named this T-DNA insertion allele of *hda6* mutants in *ros1–1* background as *hda6–9* (Fig. 1C). The second *hda6* mutant we identified in the same genetic screen has a 13-bp deletion at the end of the first exon, which caused an alteration of the last 5 amino acids in the truncated protein and a deletion of 340 amino acids as a result of a stop codon introduced by the frame shift^16^. Here, we refer to this mutant as *hda6–10* (Fig. 1C). This mutant, like *hda6–9*, also de-repressed the expression of the *RD29A-LUC* gene and restored kanamycin resistance (Fig. 1A).

**Figure 2:**
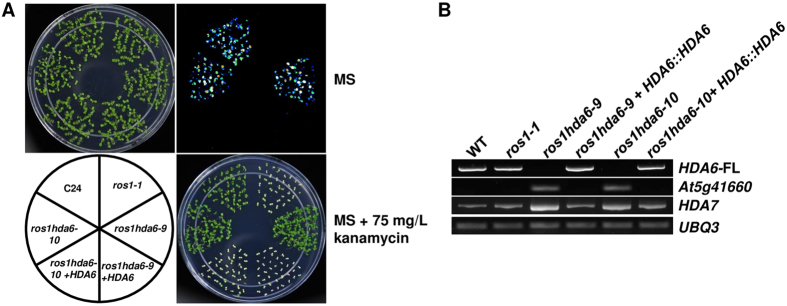
Complementation of the two hda6 mutants. (**A**) Complementation of the kanamycin resistance and luminescence expression phenotypes of the two hda6 mutants by wild-type HDA6 genomic DNA. (**B**) HDA6 genomic DNA-complemented hda6–9 and hda6–10 mutants restored ros1–1 molecular phenotypes.

We analyzed the gene expression phenotypes in these two *hda6* mutants. Unexpectedly, we found that the two mutant alleles expressed the two transgenes quite differently. Quantitative RT-PCR analysis indicated that the *hda6–10* mutant strongly released *RD29A::LUC*, with its expression level more than 10 times higher than in the *hda6–9* allele (Fig. 2A, upper panel). Consistently, the endogenous *RD29A* expression was also enhanced to a similar extent (Fig. 2A, middle panel). In contrast, the *hda6–9* allele strongly released *35S::NPTII* expression, with its expression level nearly 10 times higher than in *hda6–10* (Fig. 2A, lower panel). RNA blot analysis further confirmed the difference between the two alleles in *NPTII* expression (Fig. 2B).

Using random primers-prepared instead of oligo d(T)-synthesized cDNA library, we examined the expression of some endogenous targets of HDA6. We found that loci such as the 106B repeat sequence (centromere region), the 180bp satellite repeat sequence (centromere region), Amplicon 1 (a 45S rDNA, in a pericentromeric region)^28^ and solo LTR (A) (pericentromeric region) were more strongly released in *hda6–9* than in *hda6–10*. However, other HDA6 target loci such as IGN6 (intergenic noncoding region 6, located in regions rich in transposon-derived elements, siRNA production and DNA hypermethylation, a Pol V specific target), IGN15 (located in gene-rich regions with relatively few transposon-related repeats, a Pol V specific target)^11^,^29^ and TA3 (a LTR retrotransposon) were expressed at far higher levels in *hda6–10* than in *hda6–9*. On the other hand, TSI (transcriptional silencing information), G1136 (an endogenous genomic target of HDA6)^30^ and AtMU1 were strongly released with similar levels of expression in both *hda6* mutants (Fig. 2C).

Previous studies identified a null allele of *hda6*, *axe1–5*^31^, which was recently named *hda6–6*^32^. To comparing the two *hda6* alleles with the *hda6* null mutant in the same background, we crossed *hda6–6* (*axe1–5*) with *ros1–1* and obtained the *ros1 hda6–6* mutant (*axe1–5* was introduced into the *ros1* background) by genotyping. We conducted qRT-PCR analysis on the expression of the reporter genes and two endogenous targets. It was found that the two *hda6* alleles had far stronger releasing effects on *NPTII* and *SDC*, but had much lower releasing effects on *ERT7*^32^ relative to *ros1 hda6–6* (Fig. 2D).

**Figure 3:**
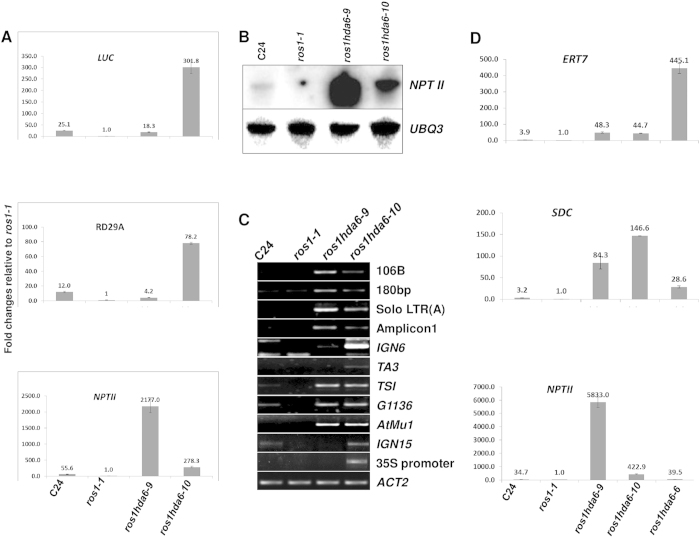
Transcriptional gene silencing is suppressed in the *hda6–9* and *hda6–10* mutant plants. (**A**) Expression levels of LUC (upper panel), endogenous *RD29A* (middle panel) and *NPTII* (lower panel) relative to those in *ros1–1* were measured with qRT-PCR using *UBQ3* as an internal reference. The Y-axis represents the fold changes compared to *ros1–1*. Ten-day-old seedlings were treated at 4 °C for 1 day, and then total RNAs were extracted for analyzing the expression of the reporter genes and endogenous genes. (**B**) RNA blot assays showed the expression level of *NPTII* in wild type, *ros1–1, hda6–9, hda6–10* and *ros1 ago4*. *UBQ3* was used as a loading control. (**C**) Expression of selected endogenous targets of HDA6 determined by RT-PCR with random hexamer primers. *ACT2* (*ACTIN2)* (its CT mean in all 4 samples with 20 cycles) was used as an internal reference. (**D**) Relative expression of Group E target *ERT7, 35S::NPTII*, and *SDC* among the two *hda6* mutated alleles and *ros1 hda6–6* (the *hda6* null mutant *axe1–5* in the *ros1–1* background). Data are means and standard derivations based on 3 biological replicates.

To confirm the suppression of transcriptional gene silencing (TGS) observed in the two *hda6* alleles was caused by the respective mutations, a genomic DNA fragment corresponding to the *HDA6* promoter and the coding region was introduced into these two mutants. The resulting transgenic plants showed kanamycin sensitivity and reduced *LUC* expression, similar to the original *ros1–1* mutant (Fig. 3A), demonstrating that the *hda6* mutations were responsible for the derepression of these transgenes silenced by the *ros1–1* mutation. RT-PCR assays demonstrated that the full-length *HDA6* transcript was detected in the transgenic plants as well as in the wild type and *ros1–1* mutant, but it was absent in the two *hda6* mutants (Fig. 3B). Interestingly, in both *hda6* mutants, there was a simultaneous increase in the expression of its close homolog *HDA7*, which likely compensated for the loss of *HDA6* (Fig. 3B). Interestingly, a *hda7* knock-down line also had a higher expression level of *HDA6* relative to the wild type^33^. This compensation effect also disappeared in the complemented lines, as indicated by an expression level of *HDA7* similar to that in the wild type and *ros1–1* (Fig. 3B). Furthermore, one of the HDA6 target genes, At5g41660, was expressed in the *hda6* mutants but was silenced again in the complemented seedlings (Fig. 3B). All these lines of evidence showed that the *hda6* mutations in the two mutants were responsible for reporter gene derepression in the *ros1–1* background.

### Differences in DNA methylation on the transgene promoters cause the opposite expression patterns of the reporter genes in the two *hda6* mutants

**Figure 4:**
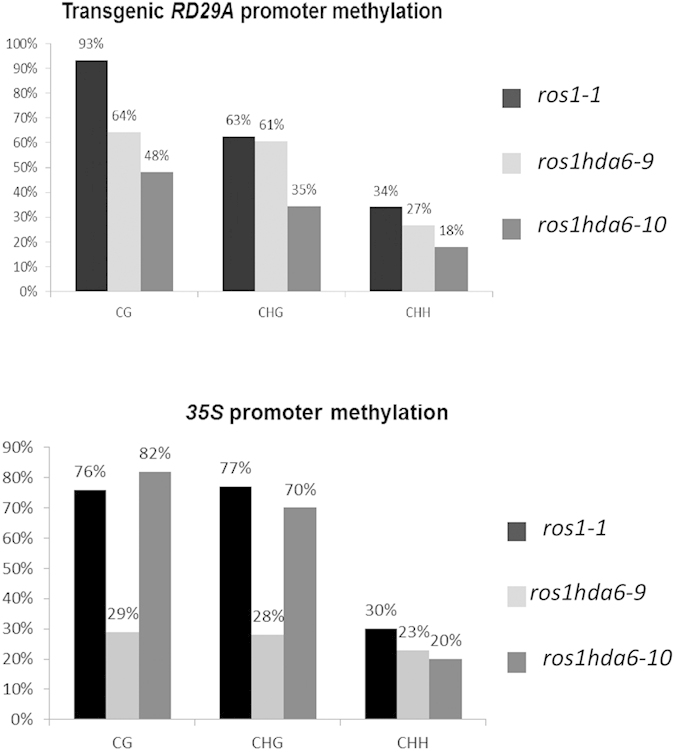
DNA methylation analysis on the *RD29A* promoter and the *35S* promoter. Bisulfite sequencing determination of DNA methylation levels on transgenic *RD29A* promoter (−270 to 0) (upper panel) and *35S* promoter (−232 to 0) (lower panel) in *ros1–1*, *hda6–9* and *hda6–10*. Data are based on 24 individual colonies for each genotype.

Transcriptional gene silencing is often caused by hypermethylation of the promoter DNA^34^. With the varied derepression of the two reporter genes in the two *hda6* alleles, we wanted to determine whether there was any difference in the methylation status of their promoters. Indeed, bisulfite sequencing showed that methylation in the CG, CHG and CHH contexts on the *35S* promoter and transgenic *RD29A* promoter had distinct differences among the genotypes (Fig. 4). Overall, the total DNA methylation in the CG, CHG and CHH contexts was lower in *hda6–10* (48%, 35% and 18%, respectively) than in *hda6–9* (64%, 61% and 27%, respectively) on the *RD29A* transgene promoter (Fig. 4A). In contrast, the CG and CHG methylation levels on the *35S* promoter were much lower in *hda6–9* (29% and 28%, respectively) than in *hda6–10* (82% and 70%, respectively). Interestingly there was a higher level of methylation in the distal region (−300 to −100) than in the proximal region (−100 to 0) of the transgenic *RD29A* promoter except for CG methylation in *ros1–1* where the methylation level was uniform across the entire promoter region (Figures S3A to S3C). A similar methylation pattern across the *35S* promoter was also found for CG and CHG methylation in the *hda6–9* mutant but not in *ros1* or *hda6–10* (Figure S4).

To corroborate the methylation status of the *35S* promoter, we conducted Chop-PCR with methylation-dependent restriction enzyme digestion that can distinguish the CG methylation status. We found that *hda6–9* had a lower DNA methylation level than the *hda6–10* mutant had, which is in accordance with our bisulfite sequencing results, although both mutants had lower DNA methylation levels than *ros1–1* and the wild type (Figure S3D). Moreover, our Chop-PCR results on Amplicon3, a nucleolar fragment in the 45S rDNA region^28^, also showed a lower CG methylation level in the *hda6–9* mutant than in the *hda6–10* mutant (Figure S3D). These data indicate that the DNA methylation levels of the two promoters inversely correlated with the gene expression levels in these alleles.

**Figure 5:**
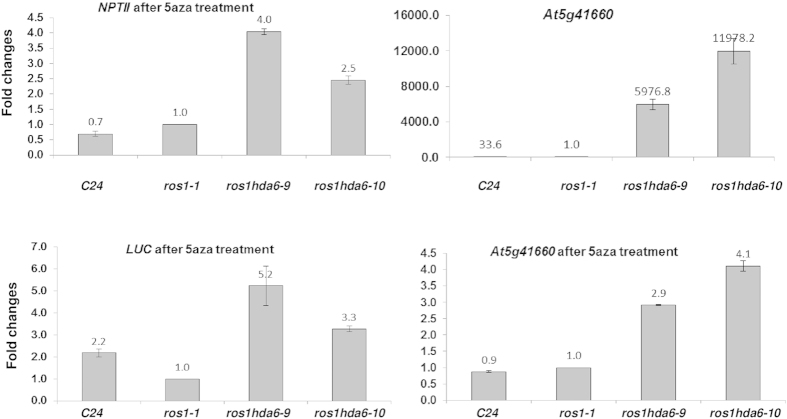
Target gene expression after 5-aza-deoxycytidine treatments. Expression levels of the indicated genes relative to those in *ros1–1* were determined with qRT-PCR using *UBQ3* as an internal reference. Data are means and standard derivations based on 3 biological replicates.

To determine whether these differences in DNA methylation contributed to the opposite effects of the two *hda6* mutations on the expression of *35S*::*NPTII* and *RD29A*::*LUC* genes, we used 5-aza-2′-deoxycytidine to block genomic DNA methylation, and checked the subsequent transcript levels of these two reporter genes. After a 14-day treatment with 5-aza-2′-deoxycytidine, the total RNA from the wild type, *ros1–1*, *hda6–9* and *hda6–10* were extracted, and qRT-PCR were performed with *NPTII, LUC* and At5g41660 (a HDA6 endogenous target gene) primers. While the 5-aza-2′-deoxycytidine treatment significantly decreased the overall methylation levels as reflected by the reporter gene expression levels, the impact on the two *hda6* alleles was striking. After the treatment, the nearly 10-fold difference in the *NPTII* expression level between *hda6–9* and *hda6–10* (Fig. 2A, lower panel) was significantly reduced to less than 2-fold (Fig. 5, left, upper panel). Similarly, the over 10 times difference in *LUC* expression between the two *hda6* mutants nearly disappeared, with the expression level in *hda6–9* even slightly higher than in *hda6–10* (Fig. 2A, upper panel and 5, left, lower panel). However, the relative expression level of At5g41660 among the two *hda6* mutants was not significantly affected by the treatment (Fig. 5, right panels). These data showed that the differing level of DNA methylation is the main reason for the distinct effects of the two *hda6* mutants on the expression pattern of *35S::NPTII* and *RD29A::LUC* genes.

### The two hda6 alleles have different abilities to interact with MET1 *in vitro* and *in vivo*

Since the two *hda6* alleles had significantly different DNA methylation patterns on the promoters of the two reporter genes as well as other target genes, we wanted to understand the molecular basis for these differences. DNA methylation is catalyzed by a group of dedicated DNA methyltransferases. Interestingly, previous research found that HDA6 and the methyltransferase MET1 shared many common targets^30^. Recently, it was reported that HDA6 in fact could physically interact with MET1^35^. We thus asked whether the two *hda6* alleles had different abilities to interact with MET1.

**Figure 6:**
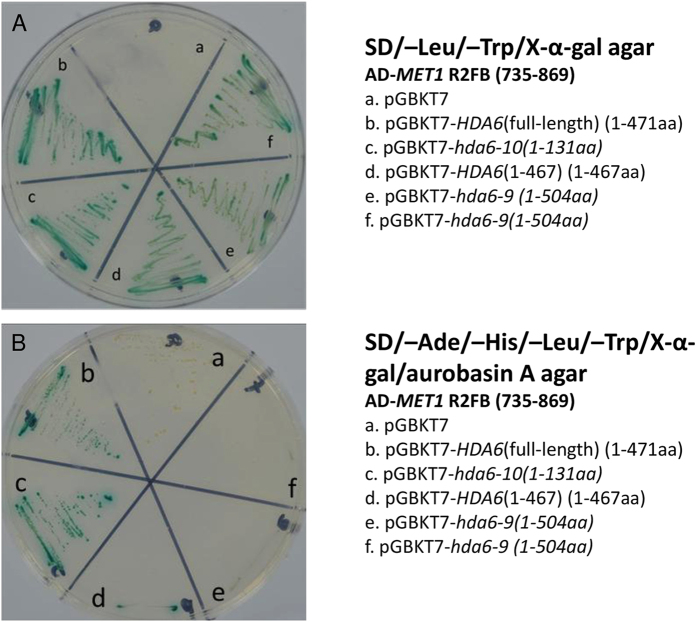
Yeast two-hybrid assays to detect the interaction between MET1 (R2FB) and hda6 mutant alleles. (**A**) Negative control (pGBKT7), wild-type HDA6 (1–471aa), mutated hda6–10 (1–131aa), HDA6 (1–467) (1–467aa) and hda6–9 (1–504aa) on the less stringent medium SD/-Trp/-Leu/X. (**B**) The interaction assay on the more stringent medium SD/-Trp/-Leu/-Ade/-His/X/A. The pictures were taken 2 days after streaking on the shown media.

We used mutated hda6 as baits and MET1 (R2FB, aa.735–869) as the prey to perform yeast two-hybrid assays. The results showed that both hda6–9 and hda6–10 could interact with MET1 *in vitro* on low stringent selective plates (SD/-Trp/-Leu/X) (Fig. 6A). However, under the higher stringent selective condition (SD/-Trp/-Leu/-Ade/-His/X/A), hda6–9 lost its ability to interact with MET1, but hda6–10 still interacted with MET1 to a similar extent as the wild-type HDA6 (Fig. 6B). These results indicated that hda6–10 has a better ability than hda6–9 in interacting with MET1 *in vitro*.

**Figure 7:**
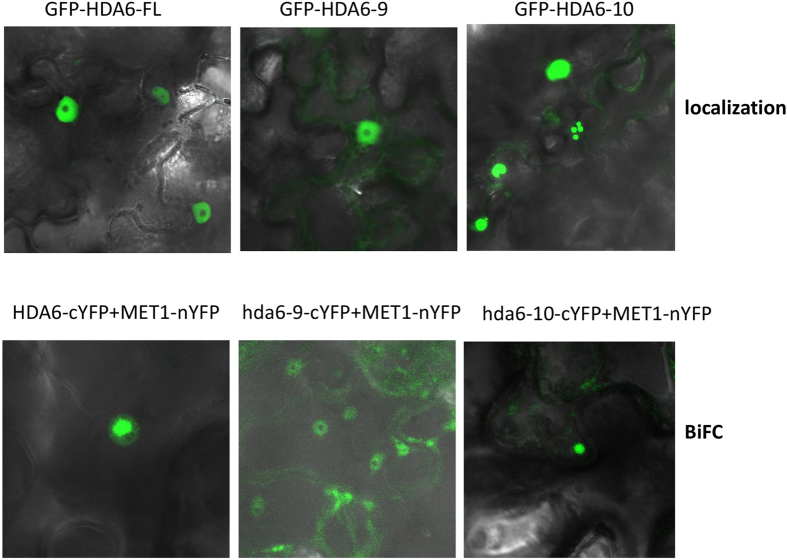
Subcellular localization of the full-length HDA6 and its mutated proteins and their *in vivo* interaction with MET1. Upper panels: Subcellular localization of the full-length HDA6 (wild type, left), hda6–9 (middle), and hda6–10 (right). Lower panels: BiFC interactions between MET1 and full-length HDA6 (wild type, left), hda6–9 (middle), and hda6–10 (right).

To investigate whether the two mutated hda6 alleles also interact with MET1 *in vivo*, we performed Bimolecular fluorescence complementation (BiFC) assays using the full-length MET1 tagged with nYFP, and the wild-type HDA6, HDA6–9, or HDA6–10 tagged with cYFP. After combining and introducing respectively the above construct pairs into the leaves of tobacco (*N. benthamiana*) via *Agrobacterium*-mediated infiltration. We found that the interaction between HDA6–9 and MET1 was far weaker than that of the wild-type HDA6 and MET1, and also weaker than that of HDA6–10 and MET1 (Fig. 7, low panel). Unlike the interaction between the wild-type HDA6 and MET1 which showed a concentrated interactive area in the nucleoplasm, or that between HDA6–10 and MET1 which showed a more limited area (strong dots in the nucleoplasm), the interaction between HDA6–9 and MET1 occurred throughout the nucleoplasm except for the nucleolus (Fig. 7 lower panel, middle). This pattern of interaction between hda6–9 and MET1 is similar to that of the localization of hda6–9 (Fig. 7, upper panel, middle).

### Histone modifications in the two *hda6* mutants

**Figure 8:**
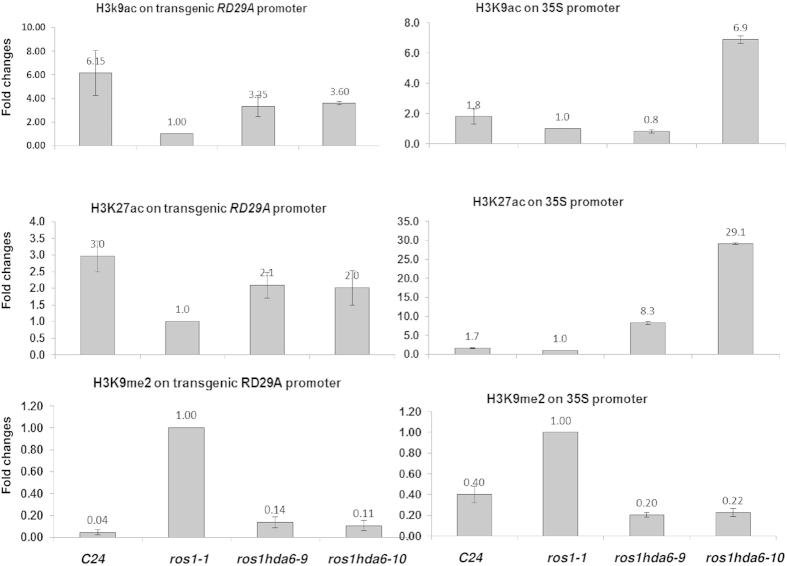
Chromatin Immunoprecipitation (ChIP) with repressive marks H3K9me2 and active marks H3K9ac and H3K27ac. H3K9me2 accumulation on the *35S* promoter (right, lower panel) and the transgenic *RD29A* promoter (left, lower panel); H3K9ac accumulation on the *35S* promoter (right, upper panel) and the *RD29A* transgene promoter (left, upper panel); H3K27ac accumulation on the *35S* promoter (right, middle panel) and the *RD29A* transgene promoter (left, middle panel). Data are means and standard derivations based on 3 technical replicates.

Chromatin status determines the accessibility of transcription factors to the target gene promoter and is regulated by DNA methylation and histone modifications. We found that the different methylation levels on the two reporter gene promoters contributed greatly to the difference in the reporter gene expression in the two *hda6* alleles. To test whether histone modifications were also involved in the different expressions of the two reporter genes in these alleles, we performed chromatin immunoprecipitation (ChIP) analysis with common repressive and active histone modification marks. The results showed that there is a higher methylation level and lower acetylation level on H3K9 in *ros1–1* compared with the wild type on all the promoters examined (Fig. 8). However, the levels of H3K9 methylation and H3K9 and H3K27 acetylation on the *RD29A* promoter were similar between the two *hda6* alleles although both alleles had higher acetylation and lower methylation levels compared with the *ros1–1* mutant. On the other hand, the levels of H3K9 and H3K27 acetylation on the *35S* promoter were quite different between the two *hda6* alleles. *hda6–10* had higher levels of H3K9 and H3K27 acetylation than *hda6–9*, *ros1–1* and the wild type (Fig. 8, right, upper and middle panels).

### Transcriptome profiles of the two *hda6* mutants reveal their differential regulation of endogenous genes

To investigate the scope of TGS in the two *hda6* alleles, we performed microarray experiments with Agilent microarrays. Compared with the *ros1–1* single mutant, 1938 targets were up regulated by more than 2-fold in *hda6–10*, and 1707 targets were up regulated in *hda6–9*. Among these up-regulated targets, 1371 were common targets shared by both *hda6* mutants (Figure S5). Although a significant number of targets were up regulated, 421 targets in *hda6–10* and 611 targets in *hda6–9* were down regulated (less than -2 fold) relative to the *ros1–1* single mutant. There were 217 common targets that were down regulated in both *hda6* mutants (Figure S5). With the two *hda6* alleles, we detected 461 targets having more than 2-fold changes in expression in *hda6–10* relative to *hda6–9* (Table S1). Among these up-regulated targets, 256 corresponded to AGI genes, 32 corresponded to non-AGI regions and 29 corresponded to transposable elements. Among down-regulated targets, 127 corresponded to AGI genes and 16 corresponded to non-AGI regions (Table S1). We also detected 321 targets having a greater than 2 or less than 2-fold change in *hda6–9* compared to *hda6–10* (Table S1).

**Figure 9:**
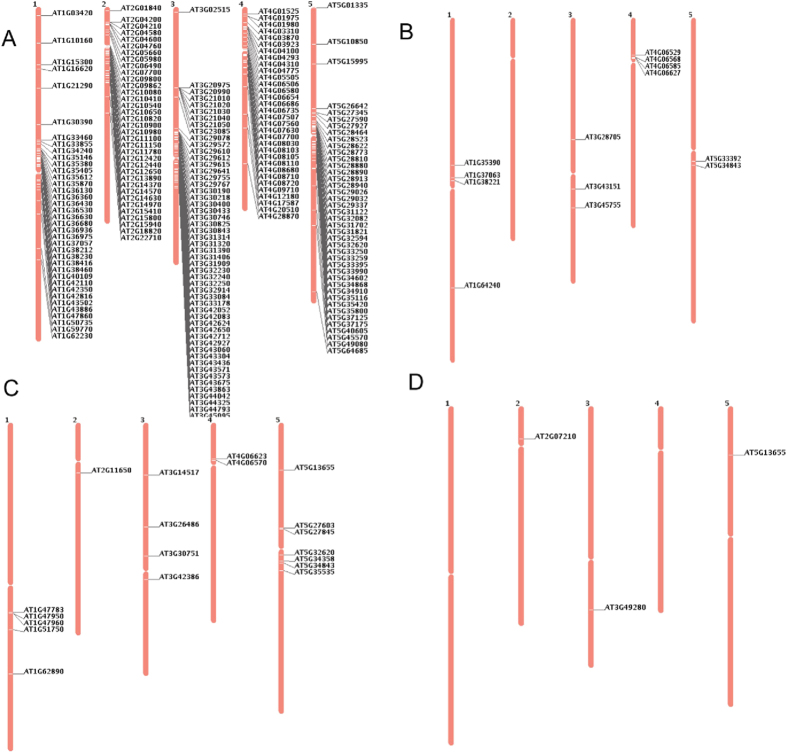
Chromosomal localization of up- or down-regulated transposons in *hda6* mutants relative to the *ros1–1* mutant. (**A**) Common up-regulated transposons in *hda6–10* and *hda6–9* relative to the *ros1–1* single mutant. (**B**) *hda6–10* specifically up-regulated transposons. (**C**) *hda6–10* specifically down-regulated transposons. (**D**) *hda6–9* specifically up-regulated transposons.

Since transposable elements are direct targets of epigenetic modifications, we tried to map to each chromosome the transposons with greater than 2-fold changes in expression in both *hda6* alleles. Our results showed that most of these up-regulated transposons were located near pericentromeric or telomeric regions (Fig. 9). In addition, some transposons were preferentially expressed in one allele relative to the other (Fig. 9B–D).

## Discussion

In a genetic screening for *ros1–1* suppressors, we identified two *hda6* alleles that suppressed *ros1–1* induced transcriptional gene silencing (TGS) of both the *RD29A::LUC* and the *35S::NPTII* reporter genes. It is known that these two reporter genes are silenced differently in the *ros1–1* mutant: the *RD29A::LUC* is silenced via the RdDM pathway in that mutants defective in the RdDM pathway all derepress the *RD29A::LUC* reporter gene but they do not show obvious derepression of the *35S::NPTII* reporter gene^8^,^15^,^16^,^17^,^36^,^37^. So far, only mutations of HDA6 and the SWI2/SNF2-like chromatin-remodeling protein DDM1 were found to derepress both the *RD29A::LUC* and the *35S::NPTII* transgenes^16^. Although it is unknown why these two reporter genes are silenced differently, notably, both DDM1 and HDA6 directly regulate the chromatin status. In this study, while it is not unexpected that the two *hda6* mutants derepressed both the *RD29A::LUC* and *35S::NPTII* reporter genes compared to the *ros1–1* mutant, surprisingly they showed distinct preferences in releasing these two reporter genes. While *hda6–9* strongly derepressed *35S::NPTII* expression, *hda6–10* strongly derepressed *RD29A::LUC* expression, suggesting that the two allelic mutations may have different impacts on DNA methylation and/or histone modifications. Interestingly, although these two *hda6* alleles had preferential releasing on transgene expression, none of them really behaved like the *hda6* null mutant *ros1 hda6–6* (axe1*–*5 in *ros1* background) (Fig. 2D). The expression level of *35S*::*NPTII* in the *ros1 hda6–6* mutant was far lower than in *hda6–9* (100 times less) or *hda6–10* (10 times less). Nonetheless, the expression of an endogenous target *ERT7* (a HDA6 and NRPE1 epistasis target)^32^ in *ros1 hda6–6* was much higher than that in *hda6–9* or *hda6–10* (nearly 10 times higher). These results show that the two hda6 alleles are weaker, rather than null, alleles of HDA6 and that they may have the ability to discriminate and work on transgenes and endogenous genes.

Our two alleles produced two different domain disrupted protein since the two mutations occurred in different domains of the HDA6 protein. The *hda6–9* mutant has a T-DNA insertion at 12 bp before the stop codon, thus causing the C-terminal disruption. On the other hand, the *hda6–10* allele has a 13-bp deletion in the first exon, which creates a premature stop codon and produces a truncated peptide lacking both the HD domain and the C-terminal domain (Fig. 1C). Given that the C-terminal domain was considered the mere interaction domain with MET1^35^, one might predict that the two *hda6* alleles should have the same or similar DNA methylation patterns since both of them have a disrupted/deleted C-terminal domain. However, the two alleles exhibited significant differences in DNA methylation at the *35S* promoter and the *RD29A* promoter, which indicated that the HD domain of HDA6 may have certain roles in DNA methylation as well. The prediction has been confirmed by a new hda6*–*8 allele which has a single amino acid substitution at ER motif of histone deacetylase domain^38^. Although yeast two-hybrid assays showed that the whole HD domain did not interact with MET1^35^, part of the HD domain of the mammalian HDAC1 (a homolog of HDA6) was found to interact with DNMT1 (a mammal homolog of MET1)^39^. Furthermore, it was reported that the single point mutation (D186N) in the HD domain of HDA6 led to reduced CG, CHG and CHH methylation levels that were even lower than in the null mutant of HDA6^40^. In addition, a point mutation (D186A) of HDA6 could not rescue the phenotypes of its *axe1–*5 mutant allele^28^. Besides the HD domain and the C-terminal domain, the N-terminal domain of HDA6 also seems to have some effects on DNA methylation, especially on rDNA repeats since the *sil1* mutant with a point mutation (Gly 16 Arg) at the N-terminus of HDA6 also caused decreased DNA methylation in the rDNA region^41^. Thus, although the C-terminus of HDA6 can physically interact with MET1, it seems that any part of the HDA6 protein is indispensable for maintaining heterochromatin status and transgene silencing via the effects on deacetylation and DNA methylation.

It is intriguing that the two *hda6* mutants would differently affect the methylation status of the two reporter genes. This may have to do, at least partly, with the difference in the interaction between the hda6 mutants and MET1, which controls much of DNA methylation. Although the two hda6 mutant proteins both interacted with MET1 on the less stringent SD/–Leu/–Trp/X media, *hda6–10* but not *hda6–9* interacted with MET1 on the more stringent SD/–Ade/–His/–Leu/–Trp/X/A media. Under these conditions, the interaction strength of *hda6–10* and MET1 was similar to if not higher than that of the wild-type HDA6 protein and MET1 (Fig. 6B). BiFC experiments using full-length MET1 tagged with the N-terminal YFP (i.e., nYFP) and wild-type HDA6 (or HDA6–9 or HDA6–10) tagged with the C-terminal YFP (i.e., cYFP) confirmed that MET1 and different HDA6 interacted *in vivo*, although the intensity of the interaction signal and patterns of interaction differed among the different HDA6 proteins (Fig. 7). The reason for the interaction between hda6–10 (a truncated protein only with N-terminal and partial HD domain) and MET1 may depend on the final 5 mutated amino acids. The wild type HDA6 without last 4 amino acid (PPSS) severely impair its interaction with MET1 (e.g hda6–9), while the last 5 mutated amino acids (VLPSN) in hda6–10 are similar to wild type last 5 amino acids (NPPSS), may thus make the truncated protein has ability to strongly interact with MET1 *in vitro*.

A previous study on the *35S::NPTII* expression in the wild type and *ros1–1* mutant showed that ROS4/IDM1, a PHD zinc-finger domain-containing histone acetyltransferase, is crucial for *NPTII* transcription^23^. The knockout line of this gene cannot release the *NPTII* expression in the *ros1–1* background even with a low DNA methylation level in the *35S* promoter^23^, demonstrating a coordinated and interdependent regulation of the *NPTII* expression by both DNA methylation and histone acetylation. In *hda6–10* mutant, although the *35S* promoter is hypermethylated (Fig. 4, lower panel), its H3K9 and H3K27 acetylation levels were higher (Fig. 8, right, upper and middle panels) than those in the other *hda6* allele, wild type as well as *ros1–1*. This enhanced histone acetylation may lead to a strong release of *NPTII* in *hda6–10* relative to *ros1–1* and the wild type despite of its hypermethylated promoter. Nonetheless, between the two *hda6* mutants, the level of histone acetylation at the examined sites (Fig. 8) did not correlate with the level of derepression of the *NPTII* gene (Figs 2C and S5). Rather, the DNA methylation levels of the *35S* promoter correlated well with the expression level of *NPTII* in the two *hda6* mutants. These data raised interesting questions regarding how the nature of these promoters affects their chromatin states and how these epigenetic modifications affect gene transcription. Previous literatures showed that heterochromatic transcription is dependent on Pol IVB/V^11^. Interestingly, the *hda6–10* mutant also showed the enhanced transcription of Pol V targets (e.g. IGN6 and IGN15, Fig 2C).

Some of the complications of differing histone acetylation among the *hda6* alleles may also have to do with their impact on the expression of other HDAs. HDA6 and HDA7 belong to the same Class I HDA1/RPD3 family^42^. HDA7 is normally not expressed in any part of the plant. However, light, heat and biotic stress could induce its expression^42^. Our data showed that HDA7 was highly expressed in the two *hda6* mutants after a 24-hour cold-treatment (Fig. 3B and S2). This compensation effect may suggest that HDA7 might play some of the histone deacetylation functions of HDA6 when HDA6 is not available. Recently, the compensation effects between *HDA6* and *HDA7* was also found in the *HDA7* knock-down line where the expression of *HDA6* and *HDA9* was significantly increased^33^.

It is known that histone modification and DNA methylation cooperatively maintain heterochromatin status to silence transgenes, transposable elements, repetitive sequences and rDNA repeats^5^,^28^,^29^. In mammalian systems, DNA methylation was thought to determine the chromatin structure via methyl DNA-specific binding proteins to recruit enzymatic machinery capable of locally altering histones^43^. On the other hand, histone modifications also determine DNA methylation via histone methylase G9a recruitment of the *de novo* DNA methylases DNMT3A and DNMT3B to methylate the underlying DNA^44^,^45^. In Arabidopsis, both the BAH domain and chromo domain of CMT3 form an aromatic cage to function as an interactive interface to capture H3K9me2 containing nucleosomes^5^. Similarly, KYP/SUVH4, a histone H3K9 methyltransferase, has a domain binding to CMT3 methylated CHG. The discoveries of different DNA methylation and histone modification of two *hda6* alleles in the current study indicate that similar coordinated functions between DNA methylation and histone modification mediated by HDA6 may also exist in plants.

## Material and Methods

### Plant growth, mutant screening and gene cloning

The Arabidopsis wild-type C24 (WT) and the *ros1–1* mutant that both harbor the homozygous *RD29A-LUC* transgene were described previously^27^. A T-DNA-mutagenized population in the *ros1–1* mutant background^26^ was used for screening for *ros1–1* suppressors. Plants were grown at 21 °C with 16 h of light and 8 h of darkness. To analyze *RD29A-LUC* expression, 7-d-old seedlings on MS plates were sprayed with luciferin (Promega) for luminescence imaging using a CCD camera as described^27^. The putative ros1–1 suppressors that emitted higher levels of luminescence than ros1–1 were transferred to soil for seed setting. These plants were further tested on Murashige and Skoog (MS) media supplement with 75 μg/ml kanamycin. The map-based cloning method was used to identify the mutated genes. The *ros1 hda6–6* double mutant was obtained by crossing *axe1–5* to *ros1–1* mutant and F_2_ population was planted on the MS agar medium supplemented with 50 μg/ml kanamycin, and the resistant seedlings were genotyped to obtain homozygous *ros1–1* and *axe1–5* seedlings.

The genomic sequence of HDA6 from wild-type C24 was amplified and cloned into the Gateway vector PMDC99 for complementation assays. The full-length *HDA6* cDNA, truncated *HDA6* cDNA (for amino acids 1–467) and mutated *hda6* cDNAs (*hda6–9* and *hda6–10*) were cloned from the wild type, *hda6–9*, and *hda6–10*, respectively. These cDNAs were further cloned into the pGBKT7 vector for yeast two-hybrid assays, and also cloned into pENTRY1A before being introduced into the binary vectors pEG104 (for localization) and pEG202-cYFP (for BiFC). The plasmid for pGADT7-MET1 (R2FB), pEG201-MET1(FL)-nYFP, and pEG202-HDA6(FL)-CYFP are kindly provided by Dr. Keqiang Wu.

### DNA methylation assays

For Chop-PCR analysis, genomic DNA was extracted using a plant DNeasy extraction kit (Qiagen) and 200 ng of genomic DNA was linearized with 20 U *Bam*HI (NEB, high fidelity) for 3 h at 37 °C. The digested DNA was purified with a Qiagen spin column and further treated with 10 U of McrBC per 100 ng of *Bam*HI-digested genomic DNA at 37 °C for 16 hours before PCR amplification. Controls also were similarly performed using H_2_O instead of McrBC. PCR amplification was conducted with Ex-Taq for 30 cycles, and the amplified fragments were analyzed on 1.7% agarose gel.

For bisulfite sequencing, 150 ng of genomic DNA were converted using a Qiagen-Epitect bisulfite kit, and the converted genomic DNA was amplified with bisulfite sequencing primers for the transgenic *RD29A* promoter, and then the amplified DNA was cloned into a pGEM-T vector (Promega). Twenty-four colonies were chosen for sequencing and DNA methylation analysis.

The 5-aza-2′-deoxycytidine treatment was performed as described^25^. Briefly, seeds were planted on MS plates supplemented with 50 μg/ml kanamycin and 7 μg/ml 5-aza-2′-deoxycytidine. Fourteen days after germination, the seedlings were treated at 4 °C for 24 h before being harvested for total RNA extraction.

### RNA analysis

For total RNA extraction, 100 mg of seedlings were harvested after cold treatment (4 °C, 1 d) and extracted using an RNeasy plant kit (Qiagen). The extracted total RNAs were treated with DNAse I and cleaned up with an RNA mini-column or treated with a Ribo-minus kit to remove rRNA from total RNA. For cDNA synthesis, 2–3 μg total RNAs with oligo d(T) or 300 ng ribo-minus RNAs with random hexamer primers were used for reverse transcription using the Invitrogen Superscript III reverse transcriptase kit. The RT solution was diluted 10 times and used for RT-PCR/qRT-PCR. For RNA blot analyses, 12 μg total RNAs from each sample were loaded, and *UBQ3* was used as an endogenous reference.

Microarray analysis was performed using the Arabidopsis Gene Expression Microarray (V4, Agilent), which contains 43,803 Arabidopsis gene probes and 1,417 Agilent control probes. There are 4 biological replicates for each sample. Total RNA (150 ng) was used to prepare Cy3-labeled probe using the low-RNA-input linear amplification/labeling kit (Agilent). The dye incorporation and copy RNA yield were measured using the Nanodrop-ND 8000 spectrophotometer (Thermo Fisher). Labeled RNA probes (1.65 mg) were fragmented using the fragmentation buffer (Agilent) and hybridized to the Arabidopsis arrays in the presence of the Gene Expression Hybridization buffer HI-RPM and the blocking agent (Agilent) for 17 h at 65 °C with a 10-rpm rotation speed in a hybridization oven (Agilent). The arrays were then washed using low stringency wash buffer 1 (Agilent) at room temperature for 1 min followed by a high-stringency wash using wash buffer 2 (Agilent) at 37 °C. The arrays were air-dried and scanned using the high-resolution array scanner (Agilent) with the appropriate settings for one-color gene expression arrays. The signal intensities were extracted from the scanned images with the aid of Feature extraction software 10.7.1.1 (Agilent) and subjected to background subtraction and spatial detrending. The outliers and the abnormal features were flagged, and the data were normalized using intraarray percentile shift normalization (threshold of 75 and above) and median-based interarray normalization. GeneSpring GX (Agilent) was used to calculate the intensity ratios and fold changes. Genes with P < 0.05 and change above 2-fold were chosen for the enrichment analysis. The microarray data were submitted to GEO(NCBI) database, the accession number is GSE73716.

### Yeast two-hybrid assays

For yeast two-hybrid assays, the Matchmaker Gold yeast two-hybrid system was used to detect the interaction between HDA6 or its mutated versions and MET1. Wild type or mutant hda6 were cloned into pGBKT7 and transformed into Y2HGold, and pGADT7-MET1 (aa. 735–869 from Dr. Keqiang Wu) was transformed into Y187. The mating and tests were done strictly following the manual provided by the manufacturer (Clontech laboratories, Inc). X-α-gal, but not X-β-gal, was used for the reporter gene assay. The lower stringent selective plates included Minimal Media Double Dropouts (SD/-Trp/-Leu) and X-α-gal, while higher stringent selective plates included Minimal Media Quadruple Dropouts (SD/-Trp/-Leu/-Ade/-His), X-α-gal, and the antibiotic Aureobasidin A.

### BiFC assays

The binary vectors harboring the wild-type HDA6 or mutant (hda6–9 or hda6–10) cDNAs tagged with C-terminal half of YFP (pEG202-HDA6/hda6–9/hda6–10-cYFP), and the binary vector harboring full-length MET1with N-terminal half of YFP (pEG201-MET1-nYFP) were introduced into GV3101 via electroporation. Single colonies of each construct were inoculated in LB medium with 50 μg/ml kanamycin, and the overnight cultures were pelleted and resuspended in a buffer (10 mM MgCl, 10 mM MES-K (pH5.6) ) supplemented with 100 μM acetosyringone, and put on bench overnight, and then the overnight cultures were Agro-infiltrated into leaves of *N. benthamiana*. Two days later, the fluorescence signals were documented with Zeiss confocal microscopy.

### Chromatin immunoprecipitation

Chromatin immunoprecipitaion assays for reporter gene promoters and a HDA6 endogenous target (At5g41660) were performed with 2-week-old seedlings. Around 1 g of fresh seedlings was harvested and cross-linked with 1% formaldehyde under vacuum for 15 minutes, and then quenched with a 2 M glycine solution and vacuumed for additional 5 minutes. After removing the formaldehyde, the sample was washed with sterilized deionized water three times. Further grinding, immunoprecipitation as well as reverse cross-linking were performed using an EpiQuick plant ChiP kit (Epigentek Inc, Farmingdale, NY) following the manual provided by the manufacturer. Anti-H3K9me2 was from Epigentek, and antibodies against H3K9ac and H3K27ac were from Millipore. The purified genomic DNA fragments were used as templates for qPCR^46^,^47^,^48^,^49^,^50^,^51^.

## Additional Information

**How to cite this article**: Zhang, S. *et al.* Two domain-disrupted hda6 alleles have opposite epigenetic effects on transgenes and some endogenous targets. *Sci. Rep.* **5**, 17832; doi: 10.1038/srep17832 (2015).

## Supplementary Material

Supplementary Information

## References

[b1] Lyko F. *et al.* Mammalian (cytosine-5) methyltransferases cause genomic DNA methylation and lethality in *Drosophila*. Nat Genet 23, 363–366 (1999). 10545955 10.1038/15551

[b2] Henderson I. R. & Jacobsen S. E. Tandem repeats upstream of the Arabidopsis endogene SDC recruit non-CG DNA methylation and initiate siRNA spreading. Genes Dev 22, 1597–1606 (2008). 18559476 10.1101/gad.1667808 PMC2428058

[b3] Cao X. & Jacobsen S. E. Locus-specific control of asymmetric and CpNpG methylation by the DRM and CMT3 methyltransferase genes. Proc Natl Acad Sci USA 99 Suppl 4, 16491–16498 (2002). 12151602 10.1073/pnas.162371599 PMC139913

[b4] Woo H. R., Dittmer T. A. & Richards E. J. Three SRA-domain methylcytosine-binding proteins cooperate to maintain global CpG methylation and epigenetic silencing in Arabidopsis. PLoS Genet 4, e1000156 (2008). 18704160 10.1371/journal.pgen.1000156 PMC2491724

[b5] Du J. *et al.* Dual binding of chromomethylase domains to H3K9me2-containing nucleosomes directs DNA methylation in plants. Cell 151, 167–180 (2012). 23021223 10.1016/j.cell.2012.07.034 PMC3471781

[b6] Stroud H., Greenberg M. V., Feng S., Bernatavichute Y. V. & Jacobsen S. E. Comprehensive analysis of silencing mutants reveals complex regulation of the Arabidopsis methylome. Cell 152, 352–364 (2013). 23313553 10.1016/j.cell.2012.10.054 PMC3597350

[b7] Mosher R. A., Schwach F., Studholme D. & Baulcombe D. C. PolIVb influences RNA-directed DNA methylation independently of its role in siRNA biogenesis. Proc Natl Acad Sci USA 105, 3145–3150 (2008). 18287047 10.1073/pnas.0709632105 PMC2268599

[b8] Gao Z. *et al.* An RNA polymerase II- and AGO4-associated protein acts in RNA-directed DNA methylation. Nature 465, 106–109 (2010). 20410883 10.1038/nature09025 PMC2865564

[b9] Lorkovic Z. J., Naumann U., Matzke A. J. & Matzke M. Involvement of a GHKL ATPase in RNA-directed DNA methylation in *Arabidopsis thaliana*. Curr Biol 22, 933–938 (2012). 22560611 10.1016/j.cub.2012.03.061

[b10] Zheng B. *et al.* Intergenic transcription by RNA polymerase II coordinates Pol IV and Pol V in siRNA-directed transcriptional gene silencing in Arabidopsis. Genes Dev 23, 2850–2860 (2009). 19948763 10.1101/gad.1868009 PMC2800093

[b11] Wierzbicki A. T., Haag J. R. & Pikaard C. S. Noncoding transcription by RNA polymerase Pol IVb/Pol V mediates transcriptional silencing of overlapping and adjacent genes. Cell 135, 635–648 (2008). 19013275 10.1016/j.cell.2008.09.035 PMC2602798

[b12] Chan S. W. *et al.* RNAi, DRD1, and histone methylation actively target developmentally important non-CG DNA methylation in arabidopsis. PLoS Genet 2, e83 (2006). 16741558 10.1371/journal.pgen.0020083 PMC1472700

[b13] Ausin I., Mockler T. C., Chory J. & Jacobsen S. E. IDN1 and IDN2 are required for *de novo* DNA methylation in Arabidopsis thaliana. Nat Struct Mol Biol 16, 1325–1327 (2009). 19915591 10.1038/nsmb.1690 PMC2842998

[b14] Zhang H., Zhu J. K. RNA-directed DNA methylation. Curr Opin Plant Biol 14, 142–147 (2011). 21420348 10.1016/j.pbi.2011.02.003 PMC3096526

[b15] Zheng Z. *et al.* An SGS3-like protein functions in RNA-directed DNA methylation and transcriptional gene silencing in Arabidopsis. Plant J 62, 92–99 (2010). 20059743 10.1111/j.1365-313X.2010.04130.x PMC2858770

[b16] He X. J. *et al.* NRPD4, a protein related to the RPB4 subunit of RNA polymerase II, is a component of RNA polymerases IV and V and is required for RNA-directed DNA methylation. Genes Dev 23, 318–330 (2009a). 19204117 10.1101/gad.1765209 PMC2648547

[b17] He X. J. *et al.* An effector of RNA-directed DNA methylation in arabidopsis is an ARGONAUTE 4- and RNA-binding protein. Cell 137, 498–508 (2009c). 19410546 10.1016/j.cell.2009.04.028 PMC2700824

[b18] Aufsatz W. *et al.* HDA6, a putative histone deacetylase needed to enhance DNA methylation induced by double-stranded RNA. EMBO J 21, 6832–6841 (2002). 12486004 10.1093/emboj/cdf663 PMC139084

[b19] Jones P. A. DNA methylation and cancer. Oncogene 21, 5358–5360 (2002). 12154398 10.1038/sj.onc.1205597

[b20] Lehnertz B. *et al.* Suv39h-mediated histone H3 lysine 9 methylation directs DNA methylation to major satellite repeats at pericentric heterochromatin. Curr Biol 13, 1192–1200 (2003). 12867029 10.1016/s0960-9822(03)00432-9

[b21] Johnson L., Cao X. & Jacobsen S. Interplay between two epigenetic marks. DNA methylation and histone H3 lysine 9 methylation. Curr Biol 12, 1360–1367 (2002). 12194816 10.1016/s0960-9822(02)00976-4

[b22] Qian W. *et al.* A histone acetyltransferase regulates active DNA demethylation in Arabidopsis. Science 336, 1445–1448 (2012). 22700931 10.1126/science.1219416 PMC3575687

[b23] Li X. *et al.* Antisilencing role of the RNA-directed DNA methylation pathway and a histone acetyltransferase in Arabidopsis. Proc Natl Acad Sci USA 109, 11425–11430 (2012). 22733760 10.1073/pnas.1208557109 PMC3396497

[b24] Zhu J. Kapoor A., Sridhar V. V., Agius F. & Zhu J. K. The DNA glycosylase/lyase ROS1 functions in pruning DNA methylation patterns in Arabidopsis. Curr Biol 17, 54–59 (2007). 17208187 10.1016/j.cub.2006.10.059

[b25] Gong Z. *et al.* ROS1, a repressor of transcriptional gene silencing in Arabidopsis, encodes a DNA glycosylase/lyase. Cell 111, 803–814 (2002). 12526807 10.1016/s0092-8674(02)01133-9

[b26] Kapoor A. *et al.* Mutations in a conserved replication protein suppress transcriptional gene silencing in a DNA-methylation-independent manner in Arabidopsis. Curr Biol 15, 1912–1918 (2005). 16271867 10.1016/j.cub.2005.09.013

[b27] Ishitani M., Xiong L., Stevenson B. & Zhu J. K. Genetic analysis of osmotic and cold stress signal transduction in Arabidopsis: interactions and convergence of abscisic acid-dependent and abscisic acid-independent pathways. Plant Cell 9, 1935–1949 (1997). 9401119 10.1105/tpc.9.11.1935 PMC157048

[b28] Earley K. *et al.* Mechanisms of HDA6-mediated rRNA gene silencing: suppression of intergenic Pol II transcription and differential effects on maintenance versus siRNA-directed cytosine methylation. Genes Dev 24, 1119–1132 (2010). 20516197 10.1101/gad.1914110 PMC2878650

[b29] Earley K. *et al.* Erasure of histone acetylation by Arabidopsis HDA6 mediates large-scale gene silencing in nucleolar dominance. Genes Dev 20, 1283–1293 (2006) 16648464 10.1101/gad.1417706 PMC1472903

[b30] To T. K. *et al.* Arabidopsis HDA6 regulates locus-directed heterochromatin silencing in cooperation with MET1. PLoS Genet 7, e1002055 (2011). 21552333 10.1371/journal.pgen.1002055 PMC3084210

[b31] Murfett J., Wang X. J., Hagen G. & Guilfoyle T. J. Identification of Arabidopsis histone deacetylase HDA6 mutants that affect transgene expression. Plant Cell 13, 1047–1061 (2001). 11340181 10.1105/tpc.13.5.1047 PMC135561

[b32] Blevins T. *et al.* A two-step process for epigenetic inheritance in Arabidopsis. Mol Cell 54, 30–42 (2014). 24657166 10.1016/j.molcel.2014.02.019 PMC3988221

[b33] Cigliano R. A. *et al.* Histone deacetylase AtHDA7 is required for female gametophyte and embryo development in Arabidopsis. Plant Physiol 163, 431–440 (2013). 23878078 10.1104/pp.113.221713 PMC3762662

[b34] Agius F., Kapoor A. & Zhu J. K. Role of the Arabidopsis DNA glycosylase/lyase ROS1 in active DNA demethylation. Proc Natl Acad Sci USA 103, 11796–11801 (2006). 16864782 10.1073/pnas.0603563103 PMC1544249

[b35] Liu X. *et al.* HDA6 directly interacts with DNA methyltransferase MET1 and maintains transposable element silencing in Arabidopsis. Plant Physiol 158, 119–129 (2012). 21994348 10.1104/pp.111.184275 PMC3252112

[b36] Liu J. *et al.* An atypical component of RNA-directed DNA methylation machinery has both DNA methylation-dependent and -independent roles in locus-specific transcriptional gene silencing. Cell Res 21, 1691–1700 (2011). 22064704 10.1038/cr.2011.173 PMC3357990

[b37] Zheng X., Zhu J., Kapoor A. & Zhu J. K. Role of Arabidopsis AGO6 in siRNA accumulation, DNA methylation and transcriptional gene silencing. EMBO J 26, 1691–1701 (2007). 17332757 10.1038/sj.emboj.7601603 PMC1829372

[b38] Hristova E., Fal K., Klemme L., Windels D. & Bucher E. HDA6 controls gene expression patterning and DNA methylation-independent euchromatic silencing. Plant physiol. 168(4), 1298–308 (2015). 25918117 10.1104/pp.15.00177 PMC4528735

[b39] Fuks F., Burgers W. A., Brehm A., Hughes-Davies L. & Kouzarides T. DNA methyltransferase Dnmt1 associates with histone deacetylase activity. Nat Genet 24, 88–91 (2000). 10615135 10.1038/71750

[b40] Aufsatz W., Stoiber T., Rakic B. & Naumann K. Arabidopsis histone deacetylase 6: a green link to RNA silencing. Oncogene 26, 5477–5488 (2007). 17694088 10.1038/sj.onc.1210615

[b41] Probst A. V. *et al.* Arabidopsis histone deacetylase HDA6 is required for maintenance of transcriptional gene silencing and determines nuclear organization of rDNA repeats. Plant Cell 16, 1021–1034 (2004) 15037732 10.1105/tpc.018754 PMC412874

[b42] Alinsug M. V., Yu C. W. & Wu K. Phylogenetic analysis, subcellular localization, and expression patterns of RPD3/HDA1 family histone deacetylases in plants. BMC Plant Biol 9, 37 (2009). 10.1186/1471-2229-9-37 PMC2671507 19327164

[b43] Hashimshony T., Zhang J., Keshet I., Bustin M. & Cedar H. The role of DNA methylation in setting up chromatin structure during development. Nat Genet 34, 187–192(2003). 12740577 10.1038/ng1158

[b44] Epsztejn-Litman S. *et al.* *De novo* DNA methylation promoted by G9a prevents reprogramming of embryonically silenced genes. Nat Struct Mol Biol 15, 1176–1183 (2008). 18953337 10.1038/nsmb.1476 PMC2581722

[b45] Feldman N. *et al.* G9a-mediated irreversible epigenetic inactivation of Oct-3/4 during early embryogenesis. Nat Cell Biol 8, 188–194 (2006). 16415856 10.1038/ncb1353

[b46] Bender J. DNA methylation and epigenetics. Annual review of plant biology 55, 41–68 (2004). 10.1146/annurev.arplant.55.031903.141641 15725056

[b47] Chinnusamy V. & Zhu J. K. RNA-directed DNA methylation and demethylation in plants. Sci China C Life Sci 52, 331–343 (2009). 19381459 10.1007/s11427-009-0052-1 PMC3139477

[b48] He X. J. *et al.* A conserved transcriptional regulator is required for RNA-directed DNA methylation and plant development. Genes Dev 23, 2717–2722 (2009b). 19903758 10.1101/gad.1851809 PMC2788325

[b49] Li E. Chromatin modification and epigenetic reprogramming in mammalian development. Nature reviews Genetics 3(9), 662–673 (2002). 10.1038/nrg887 12209141

[b50] Vaillant I. & Paszkowski J. Role of histone and DNA methylation in gene regulation. Curr Opin Plant Biol 10(5), 528–533 (2007). 17692561 10.1016/j.pbi.2007.06.008

[b51] Wierzbicki A. T., Ream T. S., Haag J. R. & Pikaard C. S. RNA polymerase V transcription guides ARGONAUTE4 to chromatin. Nat Genet 41, 630–634 (2009). 19377477 10.1038/ng.365 PMC2674513

