# Supplementary material for: Two domain-disrupted hda6 alleles have opposite epigenetic effects on transgenes and some endogenous targets
Source: Sci Rep. 2015 Dec 15;5:17832. doi: 10.1038/srep17832 (PMC4678874; doi:10.1038/srep17832)
Supplement: Supplementary Information [file srep17832-s1.pdf]

Two domain-disrupted hda6 alleles have opposite epigenetic effects on transgenes and some endogenous targets

Shoudong Zhang[1,5], Xiangqiang Zhan[2], Xiaoming Xu[3], Peng Cui[5], Jian-Kang Zhu[2,4,] Yiji Xia*[1] and Liming Xiong*[5]

[1] Department of Biology, Hong Kong Baptist University, Hong Kong, Hong Kong SAR, China. [2] Shanghai Center for Plant Stress Biology, Chinese Academy of Sciences, Shanghai, China. [3] College of life sciences, Nanjing Agricultural University, Nanjing, China. [4] Horticulture and Landscape Architecture, Purdue University, West Lafayette, IN 47907, USA.[5] Division of Biological and Environmental Sciences & Engineering, King Abdullah University of Science and Technology (KAUST), Thuwal, Saudi Arabia.

* To whom correspondence should be addressed. liming.xiong@kaust.edu.sa or yxia@hkbu.edu.hk

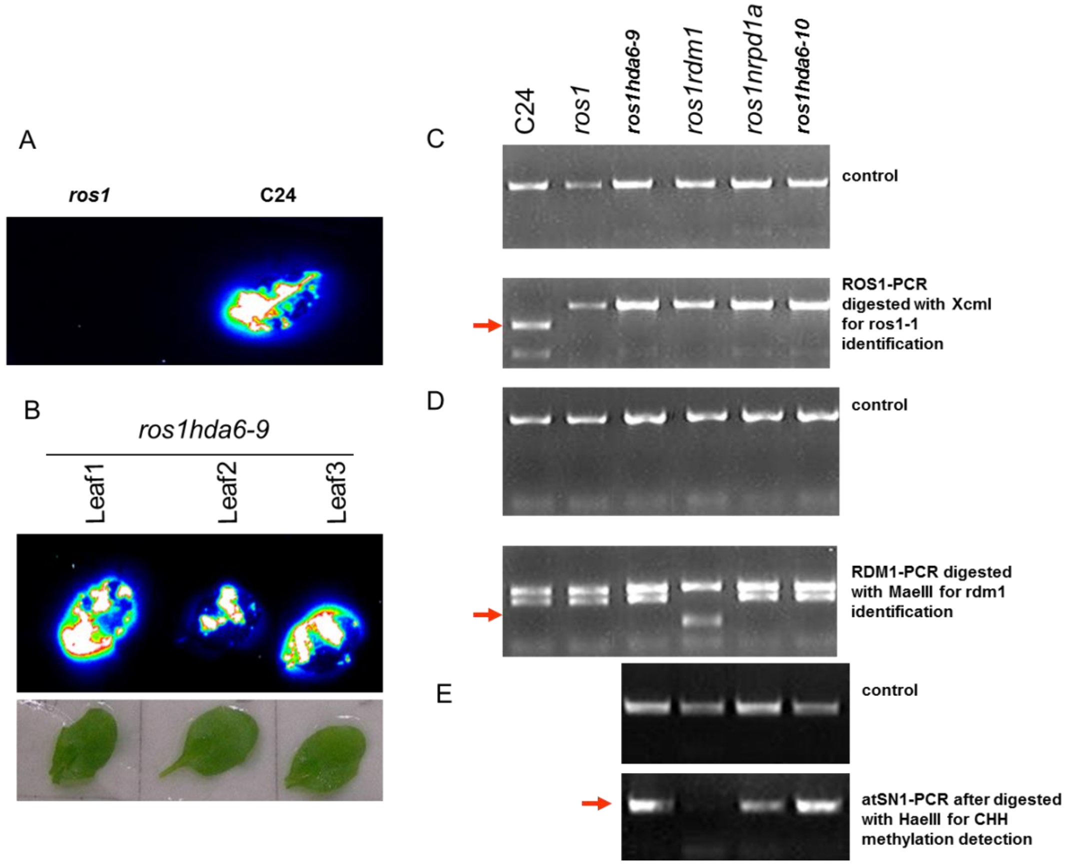

Figure S1. Identification and characterization of the *hda6-9* mutant. (A) *LUC* phenotypes of the wild type and the *ros1-1* single mutant. (B) *LUC* expression of 250 mM NaCl treated *hda6-9* leaves for 3 hours. (C) Genotyping of *hda6-9* and *hda6-10* in the *ros1-1* background. (D) Examination of possible *RDM1* mutation in the *hda6-9* and *hda6-10* mutants. (E) AtSN1 CHH methylation status of *hda6-9* and *hda6-10.*

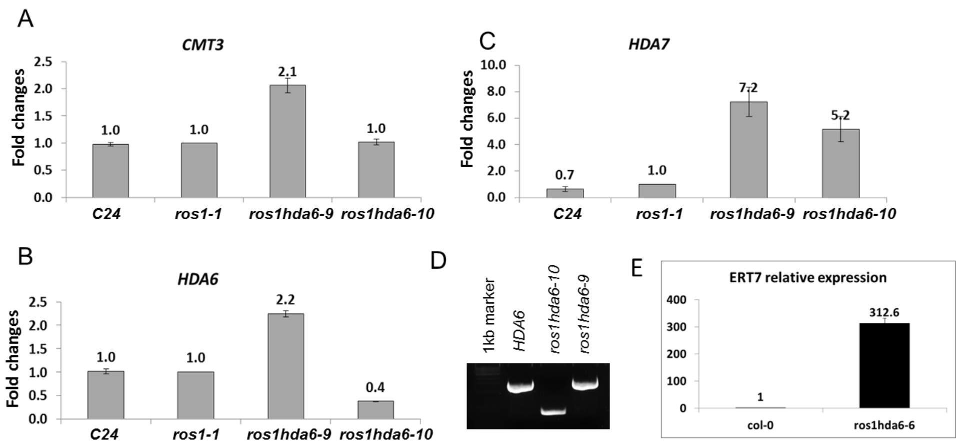

Figure S2. Expression of *CMT3*, *HDA6* and *HDA7* in *hda6* alleles. qRT-PCR detection of *CMT*3(A), *HDA6* (B) and *HDA7*(C) expression in wild type, *ros1-1*, *hda6-9* and *hda6-10*. (D) RT-PCR amplification of the full-length *HDA6* from *ros1-1*, mutated *hda6-10* from the *hda6-10* mutant, and mutated *hda6-9* from the *hda6-9* mutant. (E) RT-PCR amplification of *ERT7* from *COL-0*, mutated *ros1hda6-6*.

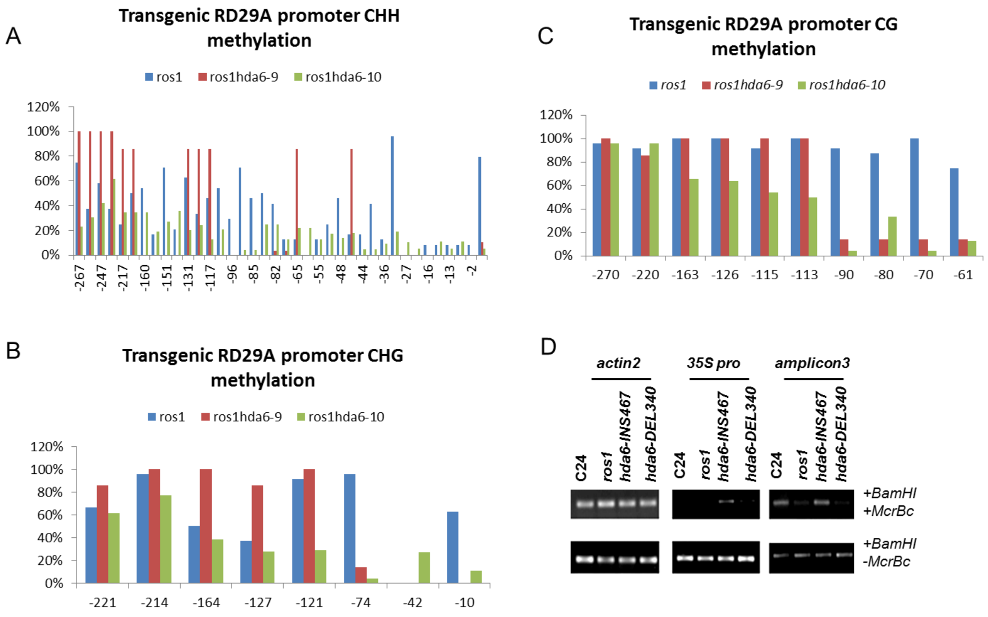

Figure S3. DNA methylation analysis on the *RD29A* promoter and the *35S* promoter. (A-C) Bisulfite sequencing to determine the transgenic *RD29A* promoter DNA methylation levels in *ros1-1*, *hda6-9* and *hda6-10* mutants. Numbers on the x-axis indicate the nucleotide positions upstream of the translation start site. (D) Chop PCR with *Bam*HI and a methylation dependent enzyme *Mcr*Bc digestion following PCR to determine the DNA methylation level.

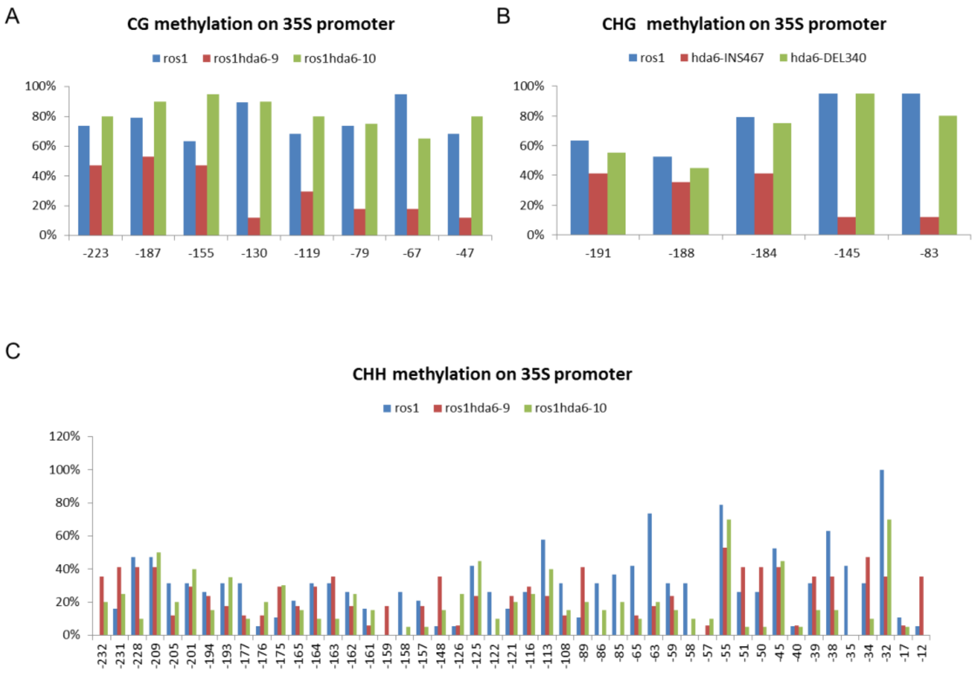

Figure S4. Bisulfite sequencing to determine the DNA methylation level of the *35S* promoter. (A) CG methylation. (B) CHH methylation. (C) CHH methylation. Numbers on the x-axis indicate the nucleotide positions upstream of the translation start site.

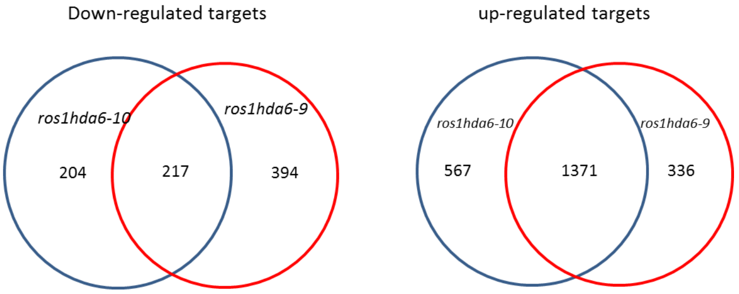

Figure S5. Comparison of up- or down- regulated targets between two *hda6* alleles relative to the *ros1-1* mutant.

Table S1. Targets with significant difference between *ros1hda6-10* and *ros1hda6-9*.

ros1hda6-10 *vs* ros1hda6-9

fold change greater than +2			fold change less than -2		
AGI gene	non-AGI	transponsable element	AGI gene	non-AGI	total
256	32	29	127	16	460

ros1hda6-9 *VS* ros1hda6-10

fold change greater than +2			fold change less than -2		
AGI gene	non-AGI	transponsable element	AGI gene	non-AGI	total
232	13	13	56	7	321

Table S2. Primers used in this study.

primers for q(RT)-PCR	qPCR-LUC-f	TGCAACTCCGATAAATAACGCGCC
	qPCR-LUC-r	AAATGTCCGTTCGGTTGGCAGAAG
	qPCR-RD29A-F	ACGACAAGAATTCTCCGATGGGCT
	qPCR-RD29A-R	TCCGGCGAATACTCGTTTCTTCCA
	qPCR-NPTII-F	tgaatgaactgcaggacgag
	qPCR-NPTII-R	atactttctcggcaggagca
	qPCR-UBQ3-F	TCTCATGCACTTGGGAGGTGAACT
	qPCR-UBQ3-R	AATACAAAGGCCCGTTACAAGCCC
	qPCR-At5g41660-F	GACGTTGCTACTCTGCCATG
	qPCR-At5g41660-R	ATTCGCCGTTGGAAGTCGTGAG
	qPCR-Actin2-F	TGCTTATGTCGCTCTTGACTAC
	qPCR-Actin2-R	CTCTCAGCTCCGATGGTTATG
	qPCR-hda7-F	GCGATTGCGGTTGGAGAACAACTT
	qPCR-hda7-R	ACGGTACACTAGGTGCGTGCATTA
	qPCR-hda6-F	GGAGACTACTACTACGGTCAAG
	qPCR-hda6-R	GGCTAGGGCGACTGATTTCTAAG
	qPCR-CMT3-f	AGACCTGCGACAAAGGATGACACT
	qPCR-CMT3-r	ATTGGCTCGTCTAGAAACCTCGCA
primers for RT-PCR	106B repeats-F	TTGATTGATAGATCCCTTCTGGA
	106B repeats-R	CGAGGATGGGGTAATTGAGT
	180bp-F	ACCATCAAAGCCTTGAGAAGCA
	180bp-R	CCGTATGAGTCTTTGTCTTTGTATCTTCT
	solo LTR A-F	ATCAATTATTATGTCATGTTAAAACCGATTG
	solo LTR A-R	TGTTTCGAGTTTTATTCTCTCTAGTCTTCATT
	Amplicon 1-F	CTCGAGGTTAAATGTTATTACTTGGTAAGATTCCGG
	Amplicon 1-R	TGGGTTTGTCATATTGAACGTTTGTGTTCATATCACC
	TA3 middle-F	GATTCTTACTGTAAAGAACATGGCATTGAGAGA
	TA3 middle-R	TCCAAATTTCCTGAGGTGCTTGTAACC
	TSI-F	CACTCTTGTTAATCCAAGTAGCTGACTCTCC
	TSI-R	GGGCTTTTGCCCATCTTCAATAGCT
	G1136-F	CTTCATCTCCGTACGCTCCTCA
	G1136-R	CATCTAGATATTAGCGATTCGGAAGAAG
	AtMuI-F	GTGGATATACCAAAAACACAA
	AtMuI-R	CAATGAGAAGGAGTATGGGAAGA
	IGN5B-F	CGCAGCGGAATTGACATCCTATC
	IGN5B-R	TCGGAAAGAGACTCTCCGCTAGAAA
	IGN6-F	GGGACATCTATTGGGTTTAGGCTGGATG
	IGN6-R	TTTGTAATTCTCAGTTCGGGTATCTGCTTG
	IGN15-F	CCATAGCATAGAAACTTGGCGATATATGAA
	IGN15-R	CGGAAAAGGTAAGGTGGTTGGAAAA
primers for Y2H	BD-hda6-NdeI-F	TTAAcatatgATGGAGGCAGACGAAAGC
	BD-hda6-EcoRI-R	AATTgaattcTTAAGACGATGGAGGATTCACG
	BD-hda6-INS467-EcoRI-R	AATTgaattcTTACACTAGCATCTGAATTTCATAACC
	BD-hda6-DEL340-EcorI-R	AAATTgaattcTTAACCAATAGAACCTCCGGC
	BD-hda6(1-467aa)-EcoRI-R	AATTgaattcATTCACGTCTGGCTCTGG
	AD-MET1 R2FB-NdeI-F	TTAAcatatgATGGTGGCTGTAGGTGG
	AD-MET1 R2FB-XhoI-R	AATTctcgagTGGAAGACTAAAGAATCCC
Primers for Bisulfite sequencing	RD29AtransBi-F1	ATATGATGGGTTAATAGATATGGAT
	RD29AtransBi-F2	AATATTTAGTTTTTTTGTAAATATA
	RD29AtransBi-R1	ATTCTATAATTTATATTCAACCCATATC
	RD29AtransBi-R2	AAATATTCCACATA CATAATATTCACC
	35S promoterBi-F	aGTAGAAAAGGAAGGTGGtAtTAtAAATG
	35S promoterBi-R	GGGTGGAGAGGTTATTTGGTTATG
Primers for chop-PCR	Chop-PCR-Amplicon 3-F	CGACCGGGTCCGAGGATTCG
	Chop-PCR-Amplicon 3-R	AACAGACATTGAAATGTCT
	Chop-PCR-35S promoter-F	ATCATTGCGATAAAGGAAAGG
	Chop-PCR-35S promoter-R	TGGAAGTATCACATCAATCC
	AtSN1-HaeIII-F	ACTTAATTAGCACTCAAATTAAACAAAATAAGT
	AtSN1-HaeIII-R	TTTAAACATAAGAAGAAGTTCCTTTTTCATCTAC
primers for CHIP	RD29A-chip-F	ATCAAGCCGACACAGACACGCG
	RD29A-chip-R	AACAGAGGAGGGTCTCACTAAG
	35S promoter Chip-F	GGAAAGGCTATCATTCAAGATGCCTCTGC
	35S promoter Chip-R	TGGAAGTATCACATCAATCCACTTGCTTT
primers for complementary	hda6-CP-f	ACGAAGCCCGATAATGACTG
	hda6-cp-r	GGTGAGAGTCGGTTCGGATA
primers for genotypes	ID-hda6-DEL340-F	AATGTCGGTGAGGATTGTCC
	ID-hda6-DEL340-R	TTGATAGCGATATCAGCGTCC
